# Expression of YKL-40 and MIP-1a proteins in exudates and transudates: biomarkers for differential diagnosis of pleural effusions? A pilot study

**DOI:** 10.1186/s12890-015-0144-6

**Published:** 2015-12-01

**Authors:** Tonia Adamidi, Nikolaos Soulitzis, Eirini Neofytou, Savvas Zannetos, Andreas Georgiou, Kleomenis Benidis, Alexis Papadopoulos, Nikolaos M. Siafakas, Sophia E. Schiza

**Affiliations:** Department of Thoracic Medicine, Nicosia General Hospital, Nicosia, Cyprus; Laboratory of Molecular and Cellular Pneumology, Medical School, University of Crete, Heraklion, Crete Greece; Department of HealthCare Management, Open University of Cyprus, Nicosia, Cyprus; Department of Thoracic Medicine, University Hospital of Heraklion, Heraklion, Crete Greece

**Keywords:** Tuberculosis, Lung cancer, Pneumonia, Metastatic cancer, Biomarkers

## Abstract

**Background:**

YKL-40 is an extracellular matrix glycoprotein with a significant role in tissue inflammation and remodeling. MIP-1a has chemotactic and pro-inflammatory properties, and is induced by YKL-40 in several lung disorders. The aim of this study was to determine the levels of YKL-40 and MIP-1a in blood serum and pleural fluids of various pulmonary diseases, and to evaluate their potential role as differential diagnosis biomarkers.

**Methods:**

We recruited 60 patients (age: 62.5 ± 20.6 years) with pleural effusions: 49 exudates and 11 transudates (T). Exudates were further classified based on the underlying disease: ten with tuberculosis (TB), 13 with lung cancer (LCa), 15 with metastatic cancer (MCa) of non-lung origin and 11 with parapneumonic (PN) effusions. YKL-40 and MIP-1a levels were measured by ELISA.

**Results:**

Pleural YKL-40 levels (ng/ml) were similar among all patient groups (TB: 399 ± 36, LCa: 401 ± 112, MCa: 416 ± 34, PN: 401 ± 50, T: 399 ± 42, *p* = 0.92). On the contrary, YKL-40 was significantly lower in the serum of TB patients (TB: 58 ± 22, LCa: 212 ± 106, MCa: 254 ± 140, PN: 265 ± 140, T: 229 ± 123, *p* < 0.001). Pleural MIP-1a protein levels (ng/ml) were statistically lower only in patients with LCa (TB: 25.0 ± 20.2, LCa: 7.3 ± 6.0, MCa: 16.1 ± 14.9, PN: 25.4 ± 27.9, T: 18.5 ± 7.9, *p* = 0.012), a finding also observed in serum MIP-1a levels (TB: 17.1 ± 7.6, LCa: 9.4 ± 7.0, MCa: 28.7 ± 28.7, PN: 33.3 ± 24.0, T: 22.9 ± 8.7, *p* = 0.003).

**Conclusions:**

Our data suggest that both YKL-40 and MIP-1a, particularly in serum, could prove useful for the differentiation of pleural effusions in clinical practice, especially of TB or LCa origin. However, large-scale studies are needed to validate these findings.

## Background

Pleural effusion is the most common manifestation of pleural disease and can develop as a result of over 50 different pleuropulmonary or systemic disorders [1]. Although the annual incidence of pleural effusions is difficult to assess, since pleural effusions are usually the result of an underlying disease, there are an estimated 1.5 million cases per year in the United States alone [2]. The most common causes for pleural effusions are congestive heart failure, infection (e.g., pneumonia), malignancy and pulmonary embolism. In general, the prevalence of pleural effusions in industrialized countries is approximately 320 cases per 100,000 residents, and is directly related to the prevalence of the underlying diseases [3]. For example, among countries with a high incidence of tuberculosis, the most frequent cause of pleural effusions is tuberculosis [4].

A major clinical challenge in the diagnosis and management of pleural effusions remains the differentiation between malignant and infectious effusions, using the right laboratory test leading to an accurate diagnosis, due to their different outcome and management [5]. Thus, the need for biomarkers that may help in this differentiation, in conjunction with the ones previously suggested by our group (IL-1A, IL-6, TNF) [6], is imperative.

The YKL-40 or Chitinase 3-like 1 protein is a growth factor for chondrocytes and fibroblasts. The precise role of this factor has not been clearly defined, but it seems that YKL-40 promotes fibroblast growth and is expressed by many cell types, such as synovial, smooth muscle cells, granulocytes, macrophages, liver and cancer cells, possessing a role in growth, tissue remodeling and inflammation [7]. YKL-40 levels increase during inflammation, since it plays an important role in chemiotaxis and in the accumulation and activation of cells associated with inflammation [7, 8]. While high levels in serum and tissues have been observed in many lung diseases [9, 10], only three studies have assessed this factor as a diagnostic tool in pleural effusions [11–13].

Macrophage inflammatory protein (MIP-1a) is a cytokine belonging to the subgroup of CCL chemokines. Chemokines are low molecular weight proteins that act as mediators in chemotactic migration of leukocytes. The synthesis of chemokines is inducted by several cells after the activation of inflammation. CCL chemokines are chemotactic for mononuclear cells, neutrophils and other granulocytes. MIP-1a plays a significant role in the chemotactic activity of monocytes and of mononuclear phagocytes. In addition, MIP-1a has a different effect on chemotactic T-lymphocytes, natural killer cells, cytotoxic T-cells, B-cells, basophils and eosinophils. In general, MIP-1a is induced by YKL-40 in lung inflammatory diseases [10], and is expressed at the stages of both acute and chronic inflammation [14, 15]. However, few studies have examined the role of this protein in pleural effusions [16–18].

The purpose of this research was to measure YKL-40 and MIP-1a in conjunction, in both pleural fluids and in the serum of patients with well-defined causes of pleural effusion, in order to ascertain their use in the differential diagnosis among the underlying diseases.

## Methods

### Study subjects

This retrospective study involved 60 patients with pleural effusions [49 exudates (EX) and 11 transudates (T)] (Table 1) who were hospitalized in the Respiratory Medicine Clinic of the Nicosia General Hospital between November 2012 and October 2014. For patients with exudates, pleural effusions were either parapneumonic (PN: *n* = 11) or were associated with tuberculosis (TB: *n* = 10), and malignant effusions associated either with lung cancer (LCa: *n* = 13) or with metastatic malignancies of non-lung origin (MCa: *n* = 15) (Table 2). The study protocol was approved by the Cyprus’s National Bioethics Committee and the Research Ethics Committee of the Medical School, University of Crete. All participants completed and signed a consent form.

**Table 1:** Clinical parameters and spirometric values of the exudates and transudates study groups

	Exudates (*n* = 49)	Transudates (*n* = 11)	*P*-Value
Clinical parameters			
Gender			
Male	33 (67.3 %)	7 (63.6 %)	1.00^a^
Female	16 (32.7 %)	4 (36.4 %)	
Age (mean ± SD, years)	60.1 ± 20.9	73.3 ± 15.7	0.055^b^
Smoking habit			
Current smokers	19 (38.8 %)	6 (54.5 %)	0.55^c^
Non-smokers	28 (57.1 %)	5 (45.5 %)	
Ex-smokers	2 (4.1 %)	0 (0.0 %)	
Pack-years (mean ± SD)	55.2 ± 36.8	80.0 ± 35.8	0.17^b^
Spirometric values			
FEV_1_ (% pred.)	79.8 ± 18.0	78.1 ± 19.1	0.78^b^
FVC (% pred.)	78.2 ± 15.8	76.0 ± 16.7	0.68^b^
FEV_1_/FVC (%)	79.8 ± 7.6	76.7 ± 10.1	0.25^b^

^a^Fisher’s exact test; ^b^Student’s *t*-test; ^c^Chi-square test

**Table 2:** Clinical parameters among the four exudates subgroups

	Tuberculosis (*n* = 10)	Lung Ca (*n* = 13)	Metastatic Ca (*n* = 15)	Parapneumonic effusions (*n* = 11)	*P*-value
Gender					
Male	6 (60.0 %)	11 (84.6 %)	8 (53.3 %)	8 (72.7 %)	0.32^a^
Female	4 (40.0 %)	2 (15.4 %)	7 (46.7 %)	3 (28.3 %)	
Age (mean ± SD, years)	27.1 ± 5.2	71.1 ± 13.3	70.4 ± 11.3	63.2 ± 16.5	<0.001^b^
Smoking habit					
Current smokers	0 (0.0 %)	9 (69.2 %)	5 (33.3 %)	5 (45.5 %)	0.023^a^
Non-smokers	10 (100.0 %)	3 (23.1 %)	9 (60.0 %)	6 (54.5 %)	
Ex-smokers	0 (0.0 %)	1 (7.7 %)	1 (6.7 %)	0 (0.0 %)	
Pack-years (mean ± SD)	—	57.5 ± 29.6	73.3 ± 46.8	29.0 ± 26.6	0.11^b^

^a^Chi-square test; ^b^Kruskal-Wallis H test

The determination of the etiology of pleural effusions was based on widely accepted criteria. The classification between exudates and transudates was based on Light’s criteria [19], using serum and pleural fluid total protein and LDH measurements, and was further confirmed by the clinical diagnosis. Within exudates, PN effusions were characterized by coexistence of pneumonia, response to antibiotics and/or pleural fluid neutrophilia. Malignant effusions were diagnosed by cytological or histological examination. TB effusions were diagnosed with the presence of positive stain or culture for Mycobacterium tuberculosis in the pleural fluid, sputum or pleural biopsy, or with the presence of typical caseating granulomas in pleural biopsy, adenosine deaminase levels in pleural fluid greater than 40 U/L and response to antituberculous therapy.

### Sample collection and processing

Samples were obtained during the first day of patient’s hospitalization and from the first successful thoracentesis, before patients had received any treatment. Simultaneously, 10 mL of venous blood were obtained. Samples were analyzed for total and differential cell count, glucose, total protein, LDH and pH. Additionally, cytological examinations and cultures for common pathogens and Mycobacterium tuberculosis were routinely performed in all pleural fluid samples. Aliquots of pleural fluid and blood samples were immediately centrifuged at 4000 g for 10 min at room temperature and the supernatants were stored at -80 °C until ΥΚL-40 and ΜΙΡ-1a protein measurements.

### ELISA detection

Complying with the ERS Task Force guidelines regarding immunoassays [20], initial experiments were conducted, in order to verify the validity and reproducibility of the measurements. In all cases spike recovery was above the recommended 80 %, and therefore the assays could be safely used to determine the levels of the studied molecules. Subsequently, YKL-40 and MIP-1a protein levels were determined with the Human YKL-40 Platinum ELISA kit and Human MIP-1a Platinum ELISA kit, respectively (eBioscience Inc, San Diego, CA, USA). Plates were read on ELx808™ Absorbance Microplate Reader (BioTek Instruments Inc, Winooski, VT, USA), at 450 nm, using 630 nm as reference wavelength. Each sample was measured at least four times (two wells in two different assays) in order to minimize intra- and inter-assay variations. Samples’ analysis was performed at the Molecular and Cellular Pulmonology Research Laboratory of the Medical School of the University of Crete.

### Statistical analysis

Differences in YKL-40 and MIP-1a levels between our study groups were determined using Student’s *t*-test, or its non-parametric equivalents Mann-Whitney U and Kruskal-Wallis H tests. In case of a statistically significant result, a post hoc analysis was performed to determine the pairwise differences among the groups. Pearson’s or the non-parametric Spearman’s rank correlation was used to examine their association with continuous variables (age, pack-years, spirometric values, etc). Additionally, the *χ*^2^ test was used to examine their association with the various clinical parameters after stratification. Finally, univariate general linear model analysis, with age and smoking status as co-factors, was used in order to correct the results for the differences among the study groups.

For the evaluation of the diagnostic performance of YKL-40 and MIP-1a levels, Receiver Operator Characteristics (ROC) analysis was performed for all recognised significant differences among the groups. The Area under the Curve (AUC) was calculated with 95 % Confidence Intervals (CIs). The optimal cut-off point was set as the value with the greatest sum of sensitivity and specificity. Consequently, sensitivity, specificity, Positive Likelihood Ratio (PLR), Negative Likelihood Ratio (NLR), Positive Predictive Value (PPV) and Negative Predictive Value (NPV) were calculated for each optimal point.

Statistical analyses were 2-sided and were performed with IBM SPSS Statistics v22.0 (IBM Corp, Armonk, NY, USA). Statistical significance was set at the 95 % level (*P* < 0.05). All data are presented as Mean ± Standard Deviation (SD).

### Power and sample size calculations

Power and sample size calculations were performed with the “PS Power and Sample Size Calculations” program (version 3.1.2) (http://biostat.mc.vanderbilt.edu/wiki/Main/PowerSampleSize). For sample size calculations, power (type II error, or β) was set to 80 % and statistical significance (type I error, or α) was set to 0.05. For power calculations, size was set to 12 (for each exudates subgroup) and type I error was set to 0.05. For both calculations, the meaningful differences in the mean value of YKL-40 and MIP-1a between the groups (22 ng/ml) and the maximum standard deviation (15) were used.

## Results

### Clinical data analysis

As seen in Table 1, there were no differences among the clinical or spirometric values between exudates and transudates. Additionally, there were also no significant differences in the clinical values among the 4 exudates subgroups, apart from the age (*p* < 0.001) and smoking status (*p* = 0.023) differentiation that the TB group displayed (Table 2).

### Exudates vs. Transudates

No marked differences were observed in YKL-40 serum levels (ng/ml) between exudates and transudates (Ex: 188 ± 137 vs. T: 229 ± 123, *p* = 0.41). A similar finding was measured in YKL-40 levels (ng/ml) from pleural effusions (Ex: 404 ± 59 vs. T: 399 ± 42, *p* = 0.81).

The same observations were made for MIP-1a serum (Ex: 22.2 ± 20.9 vs. T: 22.9 ± 8.7, *p* = 0.93) and pleural effusions (Ex: 19.0 ± 19.6 vs. T: 18.5 ± 7.9, *p* = 0.94) protein levels (ng/ml).

As expected, the pleural/serum ratio of both YKL-40 (Ex: 2.15, T: 1.75) and MIP-1a (Ex: 0.86, T: 0.81) were similar between the two study groups.

### Exudates subgroups

**Fig. 1:**
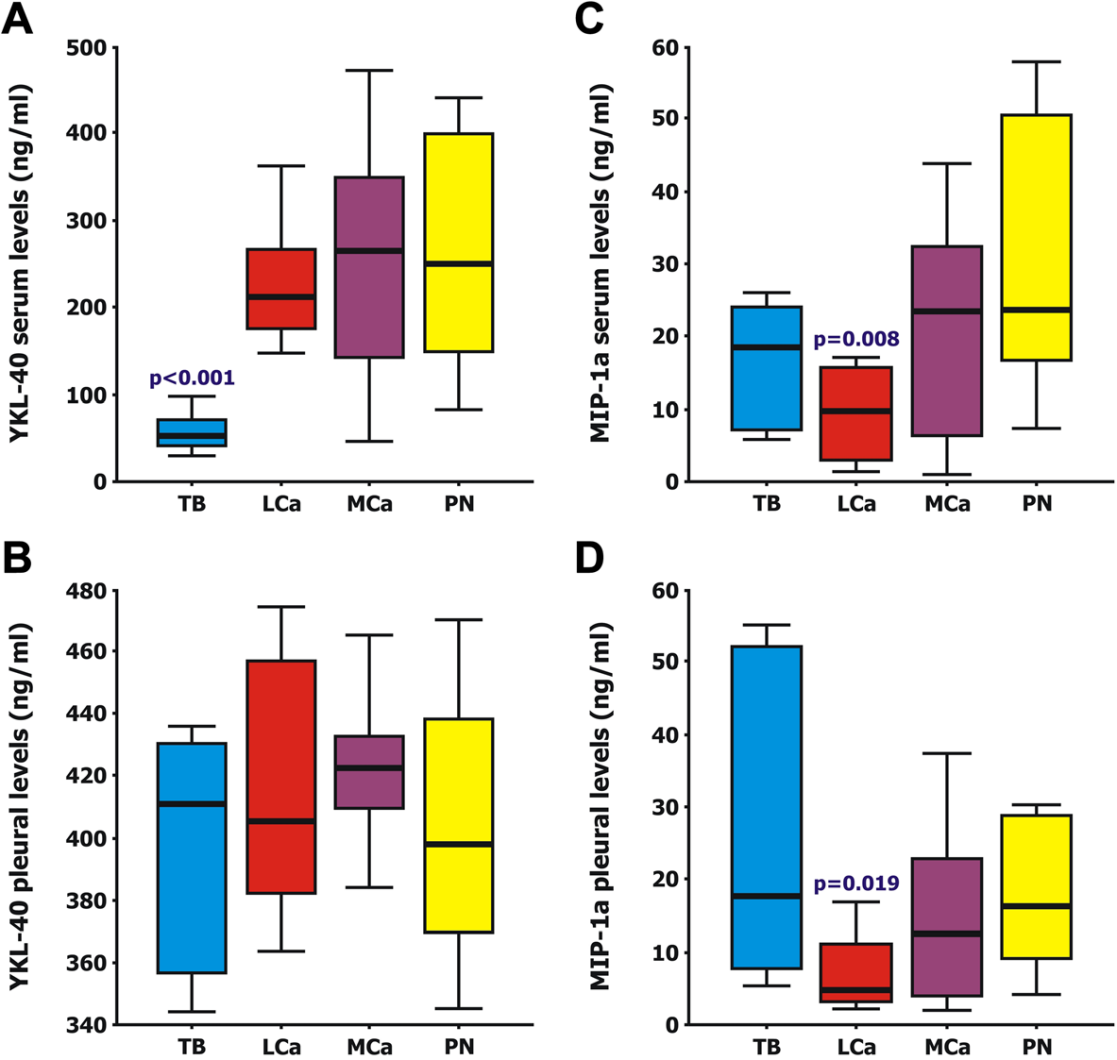
Box and whisker plots depicting the protein levels of YKL-40 (**a**, **b**) and MIP-1a (**c**, **d**) in the serum (**a**, **c**) and pleural effusions (**b**, **d**) among the 4 exudates subgroups (TB: Tuberculosis; LCa: Lung Cancer; MCa: Metastatic Cancer of non-lung origin; PN: Parapneumonic effusions)

However, when categorizing the heterogenic exudates subgroup, we found that although pleural YKL-40 levels (ng/ml) were similar among all patient groups (TB: 399 ± 36, LCa: 401 ± 112, MCa: 416 ± 34, PN: 401 ± 50, *p* = 0.85) (Fig. 1b), YKL-40 was significantly lower in the serum of TB patients (TB: 58 ± 22, LCa: 212 ± 106, MCa: 254 ± 140, PN: 265 ± 140, T: 229 ± 123, *p* < 0.001) (Fig. 1a). Subsequently, YKL-40 pleural/serum ratios were significantly higher in TB patients that in the other three exudates subgroups (TB: 6.93, LCa: 1.89, MCa: 1.64, PN: 1.52). Adenosine deaminase (ADA) levels were also high in TB patients (>40U/L). However, no correlation was found between YKL-40 serum or pleural levels with ADA expression, or with any other clinical parameters.

Because YKL-40 levels increase with age, and since TB patients were younger than the LCa, MCa and PN subgroups and displayed different smoking habits, we performed univariate general linear model analysis using age and smoking status as co-factors, in order to exclude possible biases in our findings. Even after correction, YKL-40 levels were statistically significantly lower in the serum of TB patients when compared to LCa, MCa and PN groups (*p* = 0.001).

Additionally, serum MIP-1a protein levels (ng/ml) were statistically lower only in patients with LCa (TB: 17.1 ± 7.6, LCa: 9.4 ± 7.0, MCa: 28.7 ± 28.7, PN: 33.3 ± 24.0, *p* = 0.008) (Fig. 1c), a finding also observed in pleural MIP-1a levels (TB: 25.0 ± 20.2, LCa: 7.3 ± 6.0, MCa: 16.1 ± 14.9, PN: 25.4 ± 27.9, T: 18.5 ± 7.9, *p* = 0.019) (Fig. 1d). Interestingly, the MIP-1a pleural/serum ratios were also different among the 4 exudates subgroups (TB: 1.47, LCa: 0.78, MCa: 0.56, PN: 0.76).

### Sensitivity/Specificity calculations

**Fig. 2:**
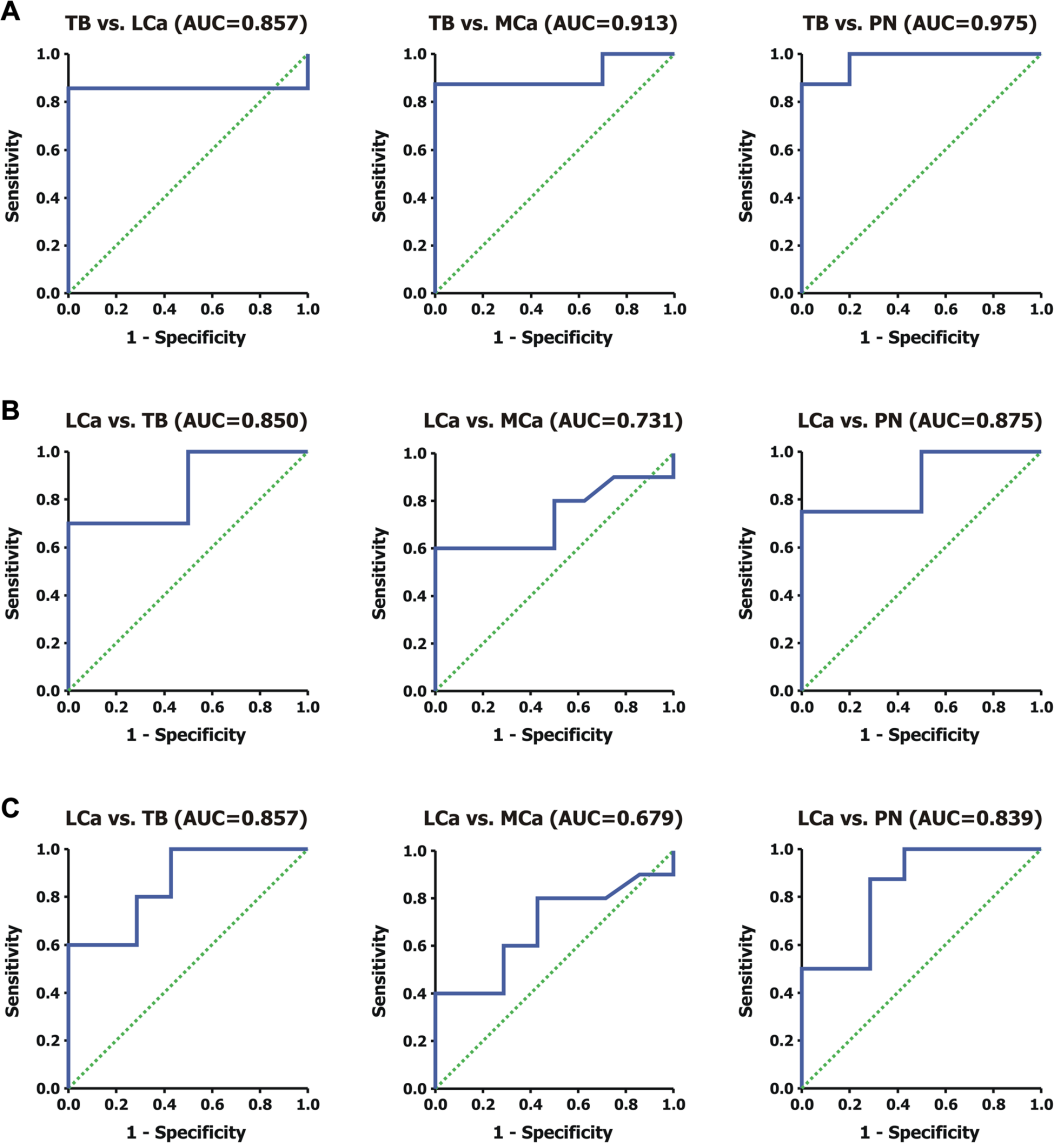
Receiver operator characteristic (ROC) analysis curves, depicting the specificity and the sensitivity of YKL-40 and MIP-1a between our study groups: **a** ROC curves of YKL-40 serum levels for the differentiation of TB vs. LCa, TB vs. MCa and TB vs. PN, respectively. **b** ROC curves of MIP-1a serum levels for the differentiation of LCa vs. TB, LCa vs. MCa and LCa vs. PN, respectively. **c** ROC curves of MIP-1a pleural levels for the differentiation of LCa vs. TB, LCa vs. MCa and LCa vs. PN, respectively. TB: Tuberculosis; LCa: Lung Cancer; MCa: Metastatic Cancer of non-lung origin; PN: Parapneumonic effusions

Using ROC analysis, we evaluated the diagnostic performance of both YKL-40 and MIP-1a proteins (Table 3). YKL-40 serum levels appear to be an excellent marker for the differentiation of tuberculosis from the other exudates. Using a cut-off point of 122.8, 118.9 and 113.2 ng/ml, it represents 91 % sensitivity and 100 % specificity for the differentiation between TB and LCa, TB and MCa, and TB and PN effusions, respectively (Table 3A, Fig. 2a).

**Table 3:** Diagnostic performance of (A) YKL-40 serum levels (ng/ml) for the differential diagnosis of Tuberculosis, (B) MIP-1a Serum levels (ng/ml) and (C) MIP-1a Pleural levels (ng/ml) for the differential diagnosis of Lung Cancer, at the optimal cut-off points of the ROC analysis

	Optimal cut off point	Sensitivity (%)	Specificity (%)	+LR	-LR	PPV (%)	NPV (%)	AUC	95 % CIs
A. YKL-40 Serum levels (ng/ml)									
TB vs LCa	122.8	90.9	100.0	>20.0	0.09	100.0	85.7	0.857	0.598–1.000
TB vs MCa	118.9	90.9	100.0	>20.0	0.09	100.0	87.5	0.913	0.745–1.000
TB vs PN	113.2	90.9	100.0	>20.0	0.09	100.0	87.5	0.975	0.913–1.000
B. MIP-1a Serum levels (ng/ml)									
LCa vs ΤΒ	22.5	100.0	70.0	3.3	>20.0	100.0	30.0	0.850	0.667–1.000
LCa vs MCa	19.8	66.7	100.0	>20.0	0.33	100.0	60.0	0.731	0.488–0.975
LCa vs PN	19.4	80.0	100.0	>20.0	0.20	100.0	75.0	0.875	0.694–1.000
C. MIP-1a Pleural levels (ng/ml)									
LCa vs TB	17.0	100.0	60.0	2.5	>20.0	100.0	60.0	0.857	0.676–1.000
LCa vs MCa	3.8	57.1	80.0	2.9	1.9	57.1	80.0	0.679	0.418–0.939
LCa vs PN	8.2	71.4	87.5	5.7	3.1	71.4	87.5	0.839	0.630–1.000

*+LR* positive likelihood ratio, *-LR* negative likelihood ratio, *PPV* positive predictive values, *NPV* Negative predictive value, *AUC* Area Under the Curve, *95 % CI* 95 % Confidence Intervals

MIP-1a serum levels (ng/ml) also distinguish LCa from the other exudates subgroups (Table 3B, Fig. 2b). For a cut-off point of 22.5 ng/ml, sensitivity is at 100 % and specificity at 70 %, for the differentiation of LCa from TB. In addition, using a cut-off point of 19.8 ng/ml, sensitivity is at 67 % and specificity at 100 %, for the differentiation of LCa and MCa. Finally, using a cut-off of 19.4 ng/ml, sensitivity is at 80 % and specificity at 100 %, for the differentiation of LCa from PN effusions. Similar results were obtained from MIP-1a pleural levels (Table 3C, Fig. 2c).

### Power calculations

According to power and sample size calculations, our study had 83.4 % power to find the statistically significant associations that were observed. Interestingly, only 12 samples on average of each exudates category were needed in order for our study to have at least 80 % power.

## Discussion

In the present study we measured the protein levels of YKL-40 and MIP-1a in pleural fluids, in order to demonstrate the correlation with their circulating levels in peripheral blood, and to determine the diagnostic value of these molecules in the differential diagnosis of pleural effusions, especially between pleural effusions associated with lung cancer and tuberculosis.

The levels of YKL-40 in pleural effusions were similar among all examined groups, without any statistical differences between them. On the contrary, YKL-40 values in the peripheral blood of patients with tuberculosis were statistical significantly lower in comparison with all the other patient categories, even after age correction. YKL-40 is expressed in the lung and serum of patients with bronchial asthma, chronic obstructive pulmonary disease, pulmonary fibrosis, sarcoidosis, lung cancer, respiratory infections, tuberculosis and cystic fibrosis [21–24].

The biological activity of YKL-40 is still largely unknown, and although a specific cell receptor for YKL-40 has not yet been found, it seems to be associated with collagen type I, II and III [25]. YKL-40 activates intracellular pathways through the cell membrane [7, 8], while acting like chitin sensor directing the macrophages and activating the anti-inflammatory response to infection [26]. Additionally, YKL-40 promotes the migration of endothelial cells and contributes to the diversification of the morphology of the endothelium. It is also found in special granules of neutrophil and mast cells [8, 27]. Nevertheless, YKL-40 inhibits oxidative damage in the lung and increases the Th2 immune response, regulates apoptosis, activates macrophages and contributes to fibrosis and rehabilitation of tissue injury [28, 29]. The expression of YKL-40 seems to be affected by IFN-γ, an important cytokine to Th1 immune response [7], while it is also activated from cytokines IL-6, IL-13, IL-17 and IL-18, which play an important role in inflammation [7, 29].

YKL-40 has not been extensively studied in pleural effusions. Kim *et al* measured the levels of YKL-40 in pleural fluid and serum of patients with tuberculosis, malignant effusions, parapneumonic effusions and transudates due to congestive heart failure [11]. Their results suggest that YKL-40 levels were higher in pleural fluids from exudates versus transudates. A similar finding was reported by Kayhan *et al* [12]. Both studies are not in accordance with our observations, in which YKL-40 levels were similar in both exudates and transudates. The high levels of YKL-40 in our transudates could be attributed to the existence of fluid in interstitial lung space, to the increased pressure in the pleural capillaries and to endothelial vessel damage, factors that can lead to increasing levels of YKL-40 [7]. In addition, patients in our study with congestive heart failure and transudates had a considerable amount of co-morbidities, such as atherosclerotic coronary artery disease, type II diabetes and smoking habit, which can also contribute to the increasing levels of YKL-40 [10, 22, 30, 31].

Moreover, in the study by Kim *et al*, YKL-40 levels were higher in pleural fluids from tuberculous pleural effusions and lower in malignant effusions [11]. In the present study the levels of YKL-40 in pleural fluids were similar among tuberculosis, lung cancer and metastatic cancer of non-lung origin. Our study, however, agrees with Kim *et al* study’s findings regarding the ratio of YKL-40 in tuberculous pleural fluid compared to that of the serum, since in both studies this percentage was higher than in the other groups [11]. This observation accounts for an important finding of our study, given that it differentiates tuberculous pleural effusions from the other exudates subgroups, with high sensitivity (91 %) and specificity (100 %), despite the significant age difference of TB patients. Although recent studies have shown that YKL-40 serum levels could be utilized in the diagnosis of endometrial carcinoma (with 74 % sensitivity and 87 % specificity) [32], and of esophageal squamous cell carcinomas (with 73 % sensitivity and 84 % specificity) [33], or could provide information regarding the response to chemotherapy and overall survival in patients with small cell lung cancer [34], this research provides evidence for the first time that YKL-40 could also be used for the differential diagnosis of tuberculosis from other pleural effusions.

The diagnostic performance of YKL-40, in comparison to already established markers like C-reactive protein (CRP) and ADA, is extremely promising. CRP had 100 % sensitivity and only 46 % specificity when distinguishing TB from malignant effusions [35], while in another study its sensitivity was 74 % and its specificity 77 %, respectively [36], findings that were verified by a third study, in which CRP performed poorly (AUC = 0.57 vs. 0.86 for YKL-40 in ours) and only ADA performed extremely well (AUC = 0.94) [37]. Another study, in which ADA levels were utilized to distinguish tuberculous from malignant effusions, had 89 % sensitivity and 70 % specificity [38], while two more studies in which TB was compared to effusions of all other origins, ADA had 87–88 % sensitivity and 92 % specificity [39, 40]. Based on the above, we can deduce that YKL-40 is superior as a diagnostic marker in distinguishing tuberculous from malignant effusions than CRP, and that is has a similar if not a better performance when compared to ADA.

Yu *et al* have shown that the immunological environment of a tuberculous pleural effusion is characterized by distinct biomarkers and in different concentrations in comparison with the serum [17]. CCL1, CCL21 factors and IL-6 are also elevated in tuberculous pleural effusions and have a specific antigenic reaction in their expression. Following the antigenic stimulation from the Mycobacterium tuberculosis, these factors are secreted in large amounts from the mononuclear cells of pleural fluid, activating YKL-40.

The anti-inflammatory protein of macrophage MIP-1a (CCL3) belongs to the cytokines family and in the subgroup of MIP-1 CC chemokines [41]. MIP-1 chemokines are produced by many cells, especially from T and B lymphocytes, neutrophils, dendritic cells, osteoblasts, astrocytes, epithelial cells of the lower airways, alveolar macrophages, eosinophils, fibroblasts and natural killer cells. The production of MIP-1 is caused by various proinflammatory factors and cytokines, such as viral infection, Gram positive bacteria, TNF-a, IFN-γ, IFN7, IL-1 α/β, IL-13 and many others [16, 41, 42]. MIP-1 chemokines act through surface receptors and while having strong chemotactic action, they play a significant action in the activation of inflammation and hemostasis [41]. Their actions include target cells via chemotaxis, degranulation, phagocytosis and mediator synthesis [41, 42]. MIP-1 chemokines play an important role in both acute and chronic inflammation, primarily with the recruitment of proinflammatory cells. Their role is particularly important in chemotaxis of T lymphocytes in the inflammatory tissues, but also in the migration of monocytes, dendritic cells and natural killer cells [42].^.^It seems that this group of chemokines, and especially MCP-1 (CCL2) whose levels increase significantly in malignant pleural effusions [16, 43–46], plays an important role in inflammatory lung diseases such as asthma, sarcoidosis, pulmonary fibrosis, but also in tuberculosis, pleural effusions, pneumonia, acute espiratory distress syndrome (ARDS) and tumors development [42, 47–49].

In our study, MIP-1a protein levels were high in all patient groups, both in the pleural fluid and the peripheral blood, with the exception of patients with malignant effusions associated with lung cancer. Their values were statistically significant lower in comparison with all the other categories (tuberculous, parapneumonic, transudates and malignant effusions associated with metastatic cancer of non-lung origin), both in the pleural fluid and the peripheral blood. In the study by Mohammed *et al*, the levels of MIP-1a were elevated in patients with complicated and non-complicated parapneumonic pleural effusions, while they were low in malignant pleural effusions and even lower in transudates associated with congestive heart failure [16]. According to that study, the chemotactic activity of MIP-1a was reduced in malignant pleural effusions compared with parapneumonic pleural effusions. In another study by Yuan *et al*, MIP-1a along with its receptor CCR1, facilitate the migration of malignant hepatoma cells through Ca^2+^ ion channels, thus playing a significant role in hepatocellular carcinoma invasion and metastasis [50]. These findings are in accordance with ours, since MIP-1a levels were higher in effusions associated with metastatic cancers of non-lung origin, compared to effusions associated with lung cancer. Our findings also suggest that MIP-1a could also be used for the differential diagnosis of lung cancer from tuberculosis (sensitivity 100 %/specificity 70 %), from parapneumonic effusions (sensitivity 80 %/specificity 100 %) and especially from metastatic tumors of non-lung origin (sensitivity 67 %/specificity 100 %), which are more difficult to distinguish. MIP-1a has not been widely used for diagnostic purposes, apart from malignant gliomas, in which MIP-1a levels provided 100 % sensitivity and 88 % specificity for the diagnosis of this tumor type versus controls [51].

YKL-40 and MIP-1a belong to the same biochemical pathway, and MIP-1a is induced by YKL-40 in lung inflammatory diseases. Letuve *et al* demonstrated that YKL-40 causes the release of three chemokines from the alveolar macrophages of smokers with or without chronic obstructive pulmonary disease (COPD). Precisely IL-8, MCP-1 and MIP-1a seem to be associated with the pathogenesis of COPD through tissue inflammation and fibrosis [10]. In the study of Sutherland *et al*, the researchers observed the inhibitory action of acidic mammalian chitinase (AMC) in the recruitment of neutrophils through MIP-1a action. They speculated that AMC and chitinase like proteins (CLP), which YKL-40 is a member of, have cross-regulatory actions. The increased expression of CLPs leads to neutrophils recruitment and causes increased secretion of MIP-1a [52].

## Conclusion

The present study measured for the first time the protein levels of YKL-40 factor in combination with the MIP-1a chemokine, both in serum and pleural fluid, exhibiting their diagnostic value in the differential diagnosis of pleural effusions. YKL-40 levels were reduced in the serum of patients with tuberculous pleurisy versus patients with malignant effusions associated with lung cancer, parapneumonic effusions, transudates and malignant effusions associated with metastatic cancer of non-lung origin. MIP-1a levels were lower both in serum and pleural fluid of patients with malignant effusions associated with lung cancer. Our results suggest that these markers could be used for the differentiation of infectious and malignant effusions in clinical practice. They could improve the differential diagnosis between the two major causes of lymphocyte-dominant pleural effusions, i.e. tuberculosis and lung cancer, and in relation to other causes of pleural effusions. Moreover, these measurements, in conjunction with other tests, could allow for the differential diagnosis between a malignant pleural effusion associated with lung cancer and a malignant effusion associated with metastatic cancer of non-lung origin.
